# The Extraction, Biosynthesis, Health-Promoting and Therapeutic Properties of Natural Flavanone Eriodictyol

**DOI:** 10.3390/nu16234237

**Published:** 2024-12-08

**Authors:** Haiaolong Yin, Yaxian Li, Yi Feng, Lei Tian, Ye Li

**Affiliations:** 1 Faculty of Food Science and Engineering, Kunming University of Science and Technology, Kunming 650500, China; 2 School of Medicine, Kunming University of Science and Technology, Kunming 650500, China

**Keywords:** eriodictyol, natural flavanones, health-promoting effects, therapeutic properties, medicine, food

## Abstract

Eriodictyol is a flavanone compound commonly found in several edible plants. Ultrasound-assisted extraction and high-performance liquid chromatography (HPLC) are commonly used methods for the separation and analysis of eriodictyol. Many studies show that some micro-organisms can produce eriodictyol as a host. What is more, eriodictyol has a wide range of health benefits, including skincare, neuroprotective, hypoglycemic, anti-inflammatory, and antioxidant activities. In addition, the therapeutic properties of eriodictyol are cardioprotective, hepatoprotective, anticancer, with protective effects on the lungs and kidneys, and so on. This review examines the extraction, biosynthesis, and health and therapeutic properties of the natural compound eriodictyol and its value in medicine and food.

## 1. Introduction

Recently, there has been a growing interest in using natural ingredients for medical treatment and health care purposes. Flavonoids are a group of compounds which are commonly found in vegetables, fruits, and medicinal plants. They can be found in different parts of the plant, such as leaves, fruits, flowers, seeds, barks, and roots [1]. Flavonoids, found in fruit, vegetables, tea, and wine, are beneficial for health [2]. Structurally, flavonoids are composed of two benzene rings (A and B rings) with phenolic hydroxyl groups, connected by a central three-carbon atom and the basic nucleus of flavonoids is 2-phenylchromone. Flavonoids are considered secondary metabolites produced by plants through long-term natural selection. Due to the presence of hydroxyl groups, flavonoids exhibit various biological activities such as antioxidant, antibacterial, antiviral, and anti-inflammatory effects [3]. Flavanone belongs to the flavonoid classes [4], and flavanones such as naringenin have also been reported to have anti-inflammatory and antioxidant pharmacological effects [5].

**Figure 1:**
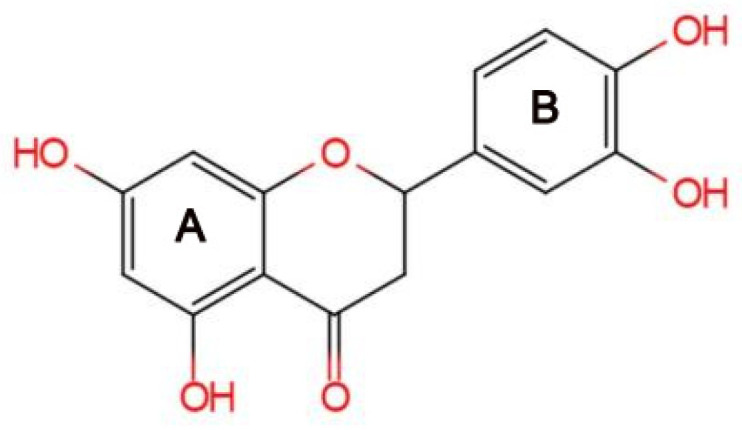
The structure of eriodictyol.

Eriodictyol is a type of flavanone compound with a chemical structure known as 2-(3,4-dihydroxyphenyl)-5,7-dihydroxy-2,3-dihydrochromen-4-one, as shown in Figure 1. It has a molecular weight of 288.25 g/mol and a molecular formula of C_15_H_12_O_6_. Numerous studies have focused on the physiological and pharmacological effects of eriodictyol, including its skin care, neuroprotective, hypoglycemic, anti-inflammatory, antioxidant, cardioprotective, hepatoprotective, anticancer, pulmonary, and renal protective effects, etc. As eriodictyol can be ingested with food, it will be increasingly important to investigate the extraction, biosynthesis, and health-promoting properties of eriodictyol in addition to its pharmacological applications. This review focuses on explaining the process of extraction and biosynthesis of eriodictyol, as well as its potential therapeutic properties/uses and health benefits.

## 2. Extraction and Biosynthesis of Eriodictyol

### 2.1. Extraction of Eriodictyol

Eriodictyol is commonly found in various edible plants such as tangerines, bitter oranges, lemons, peanuts, loquats, wormwood, oxtails, rhodiola, and so on [6]. Sato et al. showed that eriodictyol is one of the components of lemon juice and peel [7]. Miyake also proved that eriodictyol is one of the strong antioxidants in lemon fruit [8]. Gupta et al. revealed that eriodictyol is one of the citrus bioflavonoids [9]. As eriodictyol is present in foods such as citrus and lemons, this provides an opportunity for dietary supplementation with eriodictyol. It is solid at room temperature with melting point of 269–270 °C and solubility of 0.07 mg/mL at 20 °C. According to research, 12% eriodictyol can be obtained by extracting 500 g *Afzelia africana* bark with 90% methanol [10]. Lalita et al. reported that eriodictyol could be extracted from the leaves of *Anacardium occidentale* L. by ultrasound-assisted extraction, maintaining the temperature at 35 ± 5 °C using external water [11]. Moreover, HPLC analysis is a commonly used method to analyze the presence of eriodictyol in peanut shell extract [12]. In summary, eriodictyol can be extracted and identified by several phytochemical methods (Table 1).

### 2.2. Biosynthesis of Eriodictyol

Biosynthesis is another way of obtaining eriodictyol. Biosynthesis methods are more convenient and productive. Zhang et al. reported that eriodictyol could be converted from naringenin in citrus waste [52]. Patricia et al. found that the generation of genome-edited bacterial factories in *Streptomyces albidoflavus* played a role in optimizing the de novo heterologous production of eriodictyol [53]. A recent study by Wu et al. showed that using a generally safe strain of *Corynebacterium glutamicum* as a host, eriodictyol can be produced by introducing matB and matC from *Rhizobium trifolii*, which convert extracellular malonic acid to intracellular malonyl-CoA [54]. Huy et al. showed that D-glucose could serve as the initial substrate for producing eriodictyol in a co-culture *E. coli* system, and the yield of eriodictyol was evaluated by mono-culture and co-culture, respectively [55]. Brugliera et al. isolated a cDNA clone corresponding to the Ht1 locus of petunia, and found that eriodictyol could be produced by the hydroxylation of dihydrokaempferol to dihydroquercetin and naringenin, and this conversion was controlled by the locus through the flavonoid 3′-hydroxylase action [56]. Amor et al. reported that eriodictyol could be produced via the hydroxylation of naringenin at the 3′ position by the whole recombinant yeast, and they achieved the hydroxylation of naringenin through the functional expression of flavonoid 3′-hydroxylase, which was isolated from Gerbera hybrids and expressed in *Saccharomyces cerevisiae* [57]. Zhu et al. developed a method using a truncated plant flavonoid called flavonoid 3′-hydroxylase, which expresses a truncated reductase as a fusion protein in *E. coli*. By simultaneously co-expressing fusion proteins with tyrosine ammonialyase, 4-coumarate coenzyme A ligase, chalcone synthase, and chalcone isomerase, this method can be used to produce eriodictyol from l-tyrosine [58]. It can be seen that microbial biosynthesis methods were relatively effective methods for obtaining eriodictyol. In general, the details of the biosynthesis of eriodictyol are given in Table 2. This review may help people interested in the extraction and biosynthesis of eriodictyol. Extraction and biosynthesis have their own advantages; the synthesis has a higher yield, the extraction has a higher purity, and the choice depends on the raw material, the application, and the experimental or production conditions.

## 3. Health-Promoting Effects of Eriodictyol

### 3.1. Skin Care

Excessive exposure of human skin to ultraviolet A (UVA) radiation could be harmful and cause skin oxidative stress by producing reactive oxygen species (ROS), which could lead to carcinogenesis and skin aging [59]. UVA radiation can also deteriorate the collagen and elastic fibers in the dermal extracellular matrix by inducing the expression of metalloproteinase-1, thus causing damage to skin [60]. A study by Lee et al. found that eriodictyol treatment was effective in preventing the death of UV-induced keratinocytes, which are the primary cell type in the epidermis [61]. Eriodictyol inhibits the cleavage of pro-caspase-3 or pro-caspase-9 and the release of cytochrome C, and it might regulate the p38 mitogen-activated protein kinase (MAPK) and protein kinase B (AKT) signaling pathways in a phosphatase-dependent manner, thereby protecting keratinocytes from UV-induced cytotoxicity. K. Rajan et al. have suggested that eriodictyol, due to its UV filtering and free radical scavenging capabilities, can provide effective photo-protection [62]. They concluded that eriodictyol can be used in sunscreens and other cosmetics as a UV filter since it is non-toxic, non-irritating, and can absorb electromagnetic radiation in both UVA and UVB regions. Similarly, Nisar et al. found that eriodictyol can protect skin cells from oxidative damage caused by UVA radiation [63]. Their research showed that eriodictyol significantly reduced ROS production in HaCaT and FEK-4 cells when compared to the UVA-irradiated group. Eriodictyol has the potential to attenuate the expression of UVA-mediated matrix metalloproteinase-1, which could inhibit wrinkle formation or photo-aging. Additionally, eriodictyol could down-regulate the expression of some inflammatory factors in HaCaT and FEK-4 cells, including interleukin (IL)-6, IL-1β, tumor necrosis factor-α (TNF-α), transforming growth factor-β (TGF-β), cyclooxygenase-2 (COX-2), and nuclear factor kappa-B (NFκB). The intervention of eriodictyol can also inhibit the phosphorylation of MAPKs and improve the cell survival rate. It was elucidated that reducing ROS production and enhancing cell proliferation were the main reasons why eriodictyol inhibited the damage of skin cells induced by UVA radiation [63].

Excessive production of melanin in different parts of the body can lead to various skin problems such as melasma, freckles, pigmented acne scars, cancer, and senile lentigo [64]. As a result, researchers are actively seeking safe and natural melanin synthesis inhibitors to treat pigmentation [65]. Nakashima et al. reported that eriodictyol, extracted from the flower buds of *Lawsonia inermis*, effectively inhibits melanin production in theophylline-stimulated murine B16 melanoma 4A5 cells [33]. The mechanism of this inhibition is the suppression of tyrosinase, tyrosinase-related protein (TRP)-1, and TRP-2 mRNA expression by eriodictyol.

### 3.2. Neuroprotective Effects

The ethanol extract of peanut shells (PSE) has been found to contain eriodictyol, which has the potential to stimulate the differentiation of neuronal cells through the phosphoinositide 3-kinase (PI3K)-AKT and extracellular regulated protein kinase (ERK) 1/2 pathways [12]. Luteolin, eriodictyol, and 5,7-dihydroxychromone have been identified as the primary components responsible for the neurotrophic properties of PSE. Furthermore, PSE and neurotrophins synergistically promote neurite growth, indicating that PSE could be developed as a dietary supplement to potentially prevent neurodegenerative diseases such as Alzheimer’s disease (AD). In another study, it was observed that eriodictyol can protect against H_2_O_2_-induced neuron-like PC12 cell death by activating the nuclear factor erythroid 2 (Nrf2)/antioxidant response element (ARE) signaling pathway [66].

Eriodictyol has been found to have neuroprotective effects on various neurological conditions such as neuro-inflammation, oxidative stress, and synaptic dysfunction. Studies have shown that eriodictyol can alleviate lipopolysaccharide (LPS)-induced amyloidogenesis, memory impairment, and neuro-inflammation in mice and BV2 microglial cells, and promote functional recovery in spinal cord-injured rats [6,67]. Habtemariam et al. also demonstrated the neuroprotective effects of eriodictyol by targeting through Nrf2/HO-1 Axis [68]. In another study, Wang et al. discovered that eriodictyol can reduce the inflammatory response of brain injury and inhibit neuronal apoptosis by directly affecting autophagy. This research focused on the protective effect of eriodictyol on middle cerebral artery occlusion-induced brain injury in rats and its regulation of neurological function [69].

Several studies have demonstrated that eriodictyol has anti-AD properties. AD is a progressive neurodegenerative disease that is the leading cause of dementia around the world. Unfortunately, many patients lack access to effective treatments [70]. Hence, the anti-AD effect of eriodictyol is highly significant. According to a study by Jing et al., eriodictyol can protect neurons from Aβ_25–35_-induced cell death, partly by activating the Nrf2/ARE signaling pathway, and may have potential therapeutic effects for AD [71]. Additionally, Li and his team found that eriodictyol significantly improved cognitive deficits in amyloid precursor protein (APP)/presenilin 1 (PS1) mice. Eriodictyol also inhibited Aβ aggregation and Tau phosphorylation in the APP/PS1 mouse brain, and it inhibited Aβ_1–42_ oligomer-induced Tau hyperphosphorylation and neurotoxicity in HT-22 cells [72]. Furthermore, eriodictyol inhibited ferroptosis in brain cells of AD model mice, which is a new form of non-apoptotic regulated cell death that increases brain iron burden and accelerates the risk of AD progression. Eriodictyol played an anti-ferritin role in vivo and in vitro, and its mechanism may be related to the activation of the Nrf2/heme oxygenase-1 (HO-1) signaling pathway. In addition, eriodictyol and homoeriodictyol ameliorated Aβ_25–35_-induced memory impairment in mice by inhibiting NOD-like receptor pyrin domain-containing 3 (NLRP3) inflammasome, indicating that eriodictyol has potential anti-AD effects [73].

### 3.3. Hypoglycemic Effects

In previous studies, it has been suggested that eriodictyol may have anti-hyperglycemic properties. A study in diabetic rats found that eriodictyol was effective in reducing retinal levels of TNF-α, intercellular adhesion molecule-1 (ICAM-1), vascular endothelial growth factor (VEGF), and endothelial nitric oxide synthase (eNOS), which significantly improved vascular inflammation and inhibited breakdown of the blood–retinal barrier, suggesting a protective effect [74]. Lv et al. discovered that eriodictyol has a therapeutic effect on diabetic retinopathy by protecting rat RGC-5 cells from high glucose-induced oxidative stress, inflammation, and apoptosis, which achieved this by regulating the activation of the Nrf2/HO-1 pathway [75]. Eriodictyol also has the potential to treat type 2 diabetes by increasing insulin-stimulated glucose uptake in HepG2 cells and differentiated 3T3-L1 adipocytes, as well as improving insulin resistance in HepG2 cells [76]. Zeng et al. also elaborated that eriodictyol can be a potential lead compound for antidiabetic therapeutic, and identified 14 compounds, including eriodictyol, from the AcOEt fraction of *Clinopodium chinense* (BENTH) O. [19]. Additionally, it has a special glucose-dependent insulinotropic effect via the cyclic adenosine monophosphate (cAMP)/protein kinase A (PKA) pathway in mice islets [77]. Similarly, Bai et al. reported that eriodictyol increased the cell viability of mesangial cells exposed to high glucose induction and inhibited extracellular matrix accumulation, oxidative stress, and inflammation in human glomerular mesangial cells [78]. In addition, eriodictyol extracted from *Dalbergia odorifera* could be used as an α-amylase inhibitor to prevent diabetes, and eriodictyol spontaneously interacts with α-amylase at 310K and induces conformational changes of α-amylase [79]. Pérez-Ramírez et al. explained that the beneficial effects of *Flor de Junio Dalia* and *Azufrasin* cooked bean varieties on insulin resistance are related to eriodictyol [80]. A recent research has indicated that eriodictyol can also mitigate diabetic nephropathy through activating Nrf2-mediated anti-oxidative and anti-inflammatory effects [81]. A brief description of the eriodictyol hypoglycemic effect is shown in Table 3.

### 3.4. Anti-Inflammatory Effects

Eriodictyol has been found to alleviate several types of inflammation, including pneumonia, colitis, and neuroinflammation. It was observed that eriodictyol inhibited atopic dermatitis-like skin damage in ICR (Institute of Cancer Research) mice caused by 2,4-dinitrochlorobenzene, and it was also found to down-regulate the elevation of immunoglobulin E serum levels [82]. Lee et al. demonstrated that eriodictyol inhibited the release of TNFα, inducible nitric oxide synthase, IL-6, macrophage inflammatory protein-1 (MIP-1), and MIP-2 in LPS-stimulated macrophages [83]. Ferreira et al. reported that eriodictyol has the potential to prevent neuroinflammation and provide neuroprotection against permanent focal ischemia cerebral injury in male Swiss mice [84]. Imen et al. discovered that eriodictyol could reduce lysosomal enzyme activity and nitric oxide production in mouse peritoneal macrophages cultured in vitro, indicating its possible anti-inflammatory effects [85]. Mao et al. showed that eriodictyol has an anti-atherosclerotic effect by repairing damaged vascular endothelium and inhibiting the expression of inflammatory factors such as c-reactive protein, vascular endothelial growth factor, c-Jun N-terminal kinase 2, and p38 [32]. Eriodictyol could inhibit NFκB by activating the Nrf2/HO-1 signaling pathway, and showed an anti-inflammatory effect on chondrocytes that are stimulated with IL-1β [86]. Meanwhile, Liu et al. suggested that eriodictyol may be a potential treatment for rheumatoid arthritis due to its ability to inhibit inflammatory responses and rescue cell survival in RA-FLS cells by the activating the AKT/forkhead box protein O1 signaling pathway [87]. According to Wang et al., eriodictyol could attenuate the toxicity of methicillin-resistant *Staphylococcus aureus* and prevent drug resistance development by inhibiting Sortase A in mice. This suggestted that eriodictyol may potentially inhibit pneumonia caused by methicillin-resistant *Staphylococcus aureus* [88]. Hu et al. conducted a study on Wistar rats and found that eriodictyol can alleviate trinitro-benzene-sulfonic acid (TNBS)-induced ulcerative colitis by inhibiting the Toll-like receptor 4/NF-kB signaling pathway [89]. Similarly, Wang et al. found that eriodictyol can alleviate dextran sodium sulphate (DSS)-induced colitis by activating the sonic hedgehog pathway on C57BL/6 mice [90]. On the other hand, Maquera-Huacho et al. found that eriodictyol can reduce *Porphyromonas gingivalis*-induced secretion of IL-1β, IL-6, IL-8, and TNF-α in a macrophage model [91]. These anti-inflammatory effects of eriodictyol on various inflammations can be seen in Table 4.

### 3.5. Anti-Oxidative Effects

Li et al. showed that two enantiomers of eriodictyol, R-eriodictyol and S-eriodictyol, were almost equally useful in alleviating H_2_O_2_-induced oxidative stress in EA.hy926 cells [92]. Zhu et al. found that eriodictyol activated the Nrf2 pathway in an acute lung injury mouse model, which mitigated oxidative damage in macrophages [93]. Hariharan et al. showed that oral administration of eriodictyol could regulate anti-oxidative status and lipid peroxidation in albino male Wistar rats with isoproterenol-induced myocardial infarction [94]. Khlifi et al. described that heat-treated eriodictyol had the highest cellular antioxidant activity in splenocytes and macrophage cells compared to natural (non-heat-treated) molecules [95]. lv et al. demonstrated that eriodictyol decreased the emergence of ROS and enhanced the activity of catalase, glutathione peroxidase, and superoxide dismutase in high glucose-induced rat RGC-5 cells, findings suggesting that eriodictyol protected RGC-5 cells from high glucose-induced oxidative stress [75]. Vigbedor et al. found that eriodictyol isolated from the bark of *Afzelia africana* for the first time has significant anti-oxidative activity against 2,2′-azino-bis(3-ethylbenzothiazoline-6-sulfonic acid) and 2,2-Diphenyl-1-picrylhydrazyl free radicals [10]. A recent study showed that superoxide dismutase activity in HaCaT cells induced by H_2_O_2_ was significantly increased after treatment with eriodictyol, butin, butein, and liquiritigenin [96]. It was found that peanut-shell flavonoids comprising eriodictyol, luteolin, 5,7-dihydroxychromone, and eight other substances could significantly improve the antioxidant activity of sodium alginate and carrageenan composite films [97].

## 4. Potential Therapeutic Properties/Uses of Eriodictyol

### 4.1. Cardioprotective Effects

Eriodictyol shows promise as a potential treatment for cardiovascular disease and myocardial infarction. It could up-regulate the HO-1 expression in human primary endothelial cells via the ERK/Nrf2/ARE signaling pathway, which prevents oxidative stress-induced cell death [98]. They also suggested that eriodictyol has the potential to prevent cardiovascular diseases. Additionally, eriodictyol prevented hypoxia/reoxygenation-induced apoptosis of H9c2 cardiomyocytes by up-regulating the expression of B-cell lymphoma-2 (Bcl-2) and down-regulating the expression of Bcl-2-related X protein (BAX) and caspase-3, indicating that eriodictyol has the potential to treat myocardial infarction [99].

### 4.2. Pulmonary Protective Effects

Research suggests that eriodictyol has a protective effect on the lungs. Acute lung injury is a respiratory condition characterised by acute airway inflammation that can be caused by a variety of factors, including toxic inhalation pneumonitis, severe trauma, coronavirus disease 2019, acute pancreatitis, and sepsis [100,101]. Eriodictyol can inhibit the expression of inflammatory cytokines in macrophages, which contributes to its inhibitory effect on oxidative damage in mice with acute lung injury [93]. Additionally, the activation of the Nrf2 pathway by eriodictyol plays a critical role in reducing oxidative damage and protecting mice from LPS-induced acute lung injury. Eriodictyol improved LPS-induced acute lung injury by inhibiting the COX-2/NLRP3/NFκB pathway and restraining the inflammatory response [102]. He et al. demonstrated that eriodictyol can protect lung cells from alpha-hemolysin-induced injury, which is an important exotoxin in Staphylococcus aureus [103]. Eriodictyol could be used as a novel mucoregulator for inflammatory lung diseases since it inhibited phorbol 12-myristate 13-acetate-induced mucin 5AC mucin production and gene expression by inhibiting COX-2 degradation and NFκB p65 nuclear translocation [104].

### 4.3. Hepatoprotective Effects

Eriodictyol has several beneficial effects on the liver, showing improvement in hepatotoxicity, liver injury, and liver steatosis. Eriodictyol may reduce acetaminophen-induced liver damage in mice by inhibiting cytochrome P450 activity and limiting the amount of glutathione peroxidase, glutathione reductase, and glutathione S-transferase in the liver [105]. Another study found that eriodictyol protected the liver from oxidative damage caused by As_2_O_3_ in male Wistar rats by activating the Nrf2/HO-1 signaling pathway [106]. Eriodictyol can prevent the formation of fat in the liver by reducing the activity of malic enzyme, fatty acid synthase, and phosphatidate phosphohydrolase. Furthermore, eriodictyol reduced the expression of sterol regulatory element binding protein-1, acetyl-CoA carboxylase, and fatty acid synthase genes, and increased the expression of enzymes (carnitine palmitoyltransferase and β-oxidation) and peroxisome proliferator-activated receptor α gene regulating fatty acid oxidation in the liver of high-fat diet-fed mice [107]. Eriodictyol has also been shown to attenuate LPS/D-galactoamine-induced (D-GALN) acute liver injury by inhibiting apoptosis and oxidative stress through the PI3K/AKT signaling pathway [108]. It exhibits potential therapeutic effects on non-alcoholic fatty liver disease by promoting autophagy through the down-regulation of ubiquitin A-52 residue ribosomal protein fusion product 1, activating Nrf2/HO-1 to inhibit oxidative stress, and attenuate non-alcoholic fatty liver disease [109].

### 4.4. Renal Protective Effects

Eriodictyol has been shown to be protective of the kidneys. Treatment with eriodictyol reduced the production of malondialdehyde, creatinine, blood urea nitrogen, ROS, thiobarbituric acid reactive substances, TNF-α, and IL-1β in renal tissue caused by cisplatin. Additionally, eriodictyol could inhibit cisplatin-induced kidney injury in mice by activating Nrf2 and inhibiting NFκB activation [110]. The latest study by Badi et al. also showed that eriodictyol could activate the D-GALN/Nrf2 signaling pathway to reduce doxorubicin-induced nephropathy [111]. The therapeutic effects of eriodictyol on various diseases were shown in Table 5.

### 4.5. Anti-Cancer Effects

Eriodictyol has shown a potential mitigating effect on cancer; several studies have reported on its anti-cancer effects. Wang et al. found that eriodictyol showed great anticancer effects by simultaneously inducing apoptosis, G2/M cell cycle arrest, up-regulating BAX and poly ADP-ribose polymerase, and down-regulating Bcl-2 in human hepatocellular carcinoma cells [112]. Eriodictyol exhibits potential for broad application in the treatment of hepatocellular carcinoma due to its ability to suppress hepatocellular carcinoma angiogenesis, motility, cell viability, and tumor growth by deactivating the NLRP3 inflammasome [113]. Palani et al. reported that eriodictyol interacted with apoptotic proteins and may had an anti-cancer effect on colon cancer [114]. Mariyappan et al. also proved that eriodictyol could ameliorate 1,2-dimethylhydrazine-induced colon carcinogenesis in male albino Wistar rats [115]. Eriodictyol also inhibited fucosylation by down-regulating the expression of tissue-specific transplantation antigen p35b, and thus played a role in suppressing colorectal cancer [116]. In the A549 human lung cancer cell line, eriodictyol induced mitochondrial apoptosis and G2/M cell cycle arrest and inhibited mammalian target of rapamycin (mTOR)/PI3K/AKT cascade [117]. Tang et al. described that eriodictyol as a potential anti-nasopharyngeal cancer medicine by targeting the external signal-regulated kinase (MEK)/ERK signaling pathway, inducing autophagy, and hindering cell migration and invasion [118]. Eriodictyol was the most effective anti-gastric cancer flavonoid in *Polygoni orientalis* fructus, which inhibited gastric cancer cells by inhibiting the PI3K/AKT pathway [119]. Eriodictyol also modulated ferroptosis, cell viability, and mitochondrial dysfunction in ovarian cancer through Nrf2/HO-1/NADH dehydrogenase quinone 1 (NQO1) signaling pathway [120]. Eriodictyol extracted from *Polygonum perfoliatum* L. also showed potent anticancer activity [43]. Eriodictyol may inhibit the growth of lapatinib-resistant human epidermal growth factor receptor 2-positive breast cancer cells [121]. The anti-cancer effects and mechanisms of eriodictyol on various cancers are shown in Table 6.

Eriodictyol can effectively inhibit the proliferation and metastasis of glioma cell lines, U87MG and CHG-5, and induce apoptosis. This may be due to PI3K/AKT/NFκB signaling pathway inhibition [122]. Additionally, eriodictyol could inhibit the migration and invasion of glioblastoma and U87MG cell lines by down-regulating p38 MAPK/glycogen synthase kinase-3β/zinc finger E-box-binding homeobox 1 signaling pathway, thereby inhibiting the epithelial–mesenchymal transition process [123]. Liu et al. found that eriodictyol inhibited epidermal growth factor-induced neoplastic cell transformation by inhibiting ribosomal S6 kinase 2-activating transcription factor 1 signaling [124].

## 5. Other Effects

Treating human umbilical vein endothelial cells with eriodictyol led to the differential expression of 96 genes and 364 miRNAs. Eriodictyol may have a positive effect on vascular diseases through the regulation of mRNA and miRNA expression [125]. Additionally, eriodictyol could significantly promote fiber development by accumulating and maintaining the temporal auxin gradient in developing unicellular cotton [126]. In addition, Zhang et al. reported that flavonoids like eriodictyol enhance salt tolerance of plants and alleviate the adverse effects of salt stress on plants [127].

Eriodictyol is also known for its analgesic and cytotoxicity-alleviating properties. It was discovered that eriodictyol has a pain-relieving effect without the side effects and limitations expected from transient potential vanilloid 1 receptor antagonists [128]. Co-treatment with eriodictyol or naringenin can decrease benzoapyrene-induced cytotoxicity, such as cell cycle progression, oxidative stress, and apoptosis in Caco-2 cells; the protective effect of eriodictyol was found to be more significant than that of naringenin [129]. In addition, research has shown that eriodictyol is an agonist of G protein-coupled receptor 35, a substance associated with anti-inflammatory and anti-oxidative effects [130]. Eriodictyol has also been discovered to possess an antiplatelet effect, significantly inhibiting thrombin-induced platelet activation and reducing the generation of ROS in activated platelets [131].

## 6. Future Perspectives

**Figure 2:**
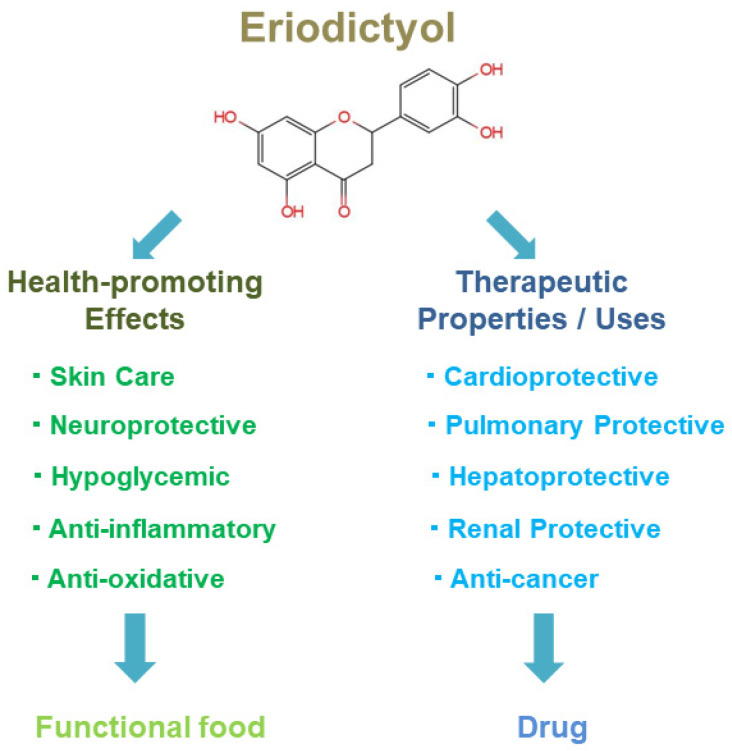
The potential applications and further use of eriodictyol.

Although there have been reviews on the pharmacological application of eriodictyol [132,133], this work has made appropriate additions from different perspectives. Eriodictyol, a natural flavanone, has been identified in various plants and has been shown in several studies to have promising skin care, neuroprotective, hypoglycaemic, anti-inflammatory, antioxidant, cardioprotective, hepatoprotective, anticancer, etc. effects (Figure 2). Researchers have identified eriodictyol as a potential candidate for the development of effective health benefits and therapeutic treatments in the future. Eriodictyol is found in some edible plants and may have a promising future in product development as a food functional factor. Moreover, further studies to elucidate the toxicology of eriodictyol in food applications are also needed. In addition, eriodictyol may be used in the development of related therapeutic agents. However, the multiple mechanisms of action of eriodictyol and its metabolites require further research.

## Figures and Tables

**Table 1:** The extraction parts and methods of eriodictyol.

Source	Tissue	Identification/Analytical Methods	Extraction Solvents/Methods	References
*Afzelia africana*	Bark	HPLC	Methanol/Maceration	[10]
*Alchemilla acutiloba*	Aerial parts	LC-ESI-MS/MS	60% methanol, diethyl ether, ethyl acetate, and n-butanol/Ultrasound-assisted	[13]
*Ampelopsis grossedentata*	Leaf	UPLC-Q-Exactive Orbitrap	Water/Maceration	[14]
*Anacardium occidentale*	Leaf	LC-DAD-MSD	80% ethanol/Ultrasound-assisted	[11]
*Arachis hypogaea*	Shell	HPLC	Ethanol/Ultrasound-assisted	[12]
*Artemisiae argyi*	Leaf	HPLC-MS/MS	Ethyl acetate/Silica gel chromatography, Sephadex LH-20 column, and preparative HPLC	[15]
*Aspalathus linearis*	Plant	UHPLC-ESI-MS	Ethanol/Ultrasound-assisted	[16]
*Asteris souliei*	Flower	HPLC	Hexane/ethyl acetate/methanol/water/Two-step high-performance counter-current chromatography method	[17]
*Citrus bergamia*	Peel	HPLC and TLC	Methanol/Ultrasound-assisted	[18]
*Clinopodium chinense* (Benth.) O. Kuntze.	Whole plant	NMR	In vitro bioactivity-guided fractionation procedure	[19]
*Coix lachryma-jobi* L. var. *ma-yuen Stapf*	Seed hull	HPLC	Ethanol/Silica gel chromatography	[20]
*Coreopsis tinctoria*	Capitula	HPLC	-	[21]
*Cyclopia subternata*	Seedling	HPLC	Water/Water bath	[22]
*Cyclotrichium origanifolium*	Aerial parts	HPLC	Hexane, ethyl acetate, and n-butanol/Maceration	[23]
*Dendrobium ellipsophyllum*	Whole plant	GC	Methanol/Chromatographic techniques including silica gel and Sephadex LH20	[24]
*Dracocephalum rupestre Hance*	Leaf	LC-MS	Methanol/Maceration	[25]
*Eurya chinensis*	Leaf	LC-MS	95% ethanol/Silica gel column chromatography	[26]
*Elsholtzia bodinieri*	Whole plant	NMR	-	[27]
*Erythrophleum Ivorense*	Root bark	HPLC-MS	Methanol/Maceration	[28]
*Feijoa sellowiana*	Fruit juice	HPLC	-	[29]
*Gleditsia sinensis*	Thorn	HPLC	Ethanol/Ultrasound-assisted	[30]
*Glycyrrhiza uralensis*	Leaf	HPLC	Ethanol/Repeated Chromatography	[31]
*Helichrysum arenarium*	Flower	HPLC	-	[32]
*Lawsonia inermis*	Flower bud	HPLC	Methanol/Extraction with ethyl acetate followed by 1-butanol	[33]
*Lophophytum*	Tuber	HPLC-MS	-	[34]
*Lyonia ovalifolia*	Aerial parts	LC-ESI-QTOF-MS/MS	Ethanol/Solvent extraction and fractionation	[35]
*Mentha pulegium*	Plant	HPLC-DAD	Water and methanol	[36]
*Mentha x villosa*	Leaf	LC-DAD	Water	[37]
Mexican Arnica	Flower	NMR	-	[38]
*Onopordum alexandrinum*	Flower	UV and NMR	Ethyl acetate fraction/Maceration	[39]
*Onosma*	Aerial part	ESI-MS/MS	Methanol/Maceration	[40]
*Passiflora subpeltata*	Fruit pulp	HPLC-MS/MS	Chloroform, acetone, and methanol/Ultrasound-assisted	[41]
*Phlomis*	Leaf	LC-MS/MS	85% methanol/Liquid Chromatogram	[42]
*Polygonum perfoliatum* L.	Stem	LC-MS/MS	95% ethanol	[43]
*Prunus persica*	Gum	LC-QQQ/MS	Air dried, ground, passed through a 60-mesh screen, and homogenized	[44]
*Scutellaria lateriflora*	Aerial parts	MS and NMR	Methanol	[45]
*Semiliquidambar chingii*	Twig	HPLC	Ethyl acetate/Reflux	[46]
*Tamarindus indica*	Shell	UPLC-MS/MS	95% ethanol/Reflux	[47]
*Thonningia sanguinea Vahl*	Plant	GC-MS/MS	Methanol/Maceration	[48]
*Thymus broussonetii*	Leaf	NMR, UV, and MS	Methanol	[49]
*Thymus species*	Exudate	GC/MS and TLC	Acetone	[50]
*Uvaria siamensis*	Stem bark	Extensive spectroscopic	-	[51]

**Table 2:** Biosynthesis of eriodictyol.

Initial Substrate	Host	Cultivation Conditions	Yield	References
Naringenin	Competent *E. coli* BL21 cells	Cultured in 5 mL of Luria–Bertani medium containing kanamycin and incubated in a shaker at 37 °C, 220 rpm for about 8 h	62.57%	[52]
*-*	*Streptomyces albidoflavus*	At 30 °C in yeast and malt extract 17% (*w*/*v*) sucrose	0.06 mg/L	[53]
Tyrosine	*Corynebacterium glutamicum*	Grown and fermented at 30 °C; stored in medium with glycerol (20%, *v*/*v*) at −80 °C for long term.	62%	[54]
D-glucose	*Escherichia coli*	Cultured in Luria–Bertani medium containing the appropriate antibiotics: ampicillin, chloramphenicol, streptomycin, or kanamycin	51.5 mg/L	[55]
Naringenin	Yeast	In a selective medium	200 mg/L	[57]
L-tyrosine	*Escherichia coli*	Inoculated in the Luria–Bertani plate and cultured at 37 °C for 12 h	107 mg/L	[58]

**Table 3:** Hypoglycemic effects of eriodictyol.

Experimental Subject	Theory or Pathway	Doses	References
Male Sprague-Dawley rats	Decreased TNFa, ICAM-1, VEGF, and eNOS	0.1, 1, and 10 mg/kg	[74]
Rat RGC-5 cells	Nrf2/HO-1 pathway	5, 10, and 20 μM	[75]
HepG2 and 3T3-L1	Glucose uptake and insulin resistance	5 and 25 μM	[76]
BALB/c mice, Wistar rats, and MIN6 cells	cAMP/PKA pathway	10 and 20 mg/kg	[77]
Human glomerular mesangial cells	Akt/NF-κB pathway	12.5 and 25 μM	[78]
α-amylase from porcine pancreas	Inhibited the activity of α-amylase	0 to 1.5 × 10^5^ mol/L	[79]

**Table 4:** Anti-inflammatory effects of eriodictyol.

Type	Experimental Subject	Inducement	References
Atopic dermatitis	Male ICR mice	2,4-dinitrochlorobenzene	[82]
Inflammation	Macrophages	LPS	[83]
Neuroinflammation	Male Swiss mice	Electrocoagulation	[84]
Inflammation	Spleen cells and macrophages	LPS	[85]
Osteoarthritis	chondrocytes	IL-1β	[86]
Rheumatoid arthritis	RA-FLSs isolated from patients	-	[87]
Pneumonia	Inbred C57BL/6J and A549 cells	*Staphylococcus aureus*	[88]
Ulcerative colitis	Wistar rats	TNBS	[89]
Colitis	C57BL/6 mice	DSS	[90]
Periodontitis	Macrophages	*Porphyromonas gingivalis*	[91]

**Table 5:** The potential therapeutic properties/uses of eriodictyol on various diseases.

Organ	Disease	Experimental Subject	Inducement	References
Heart	Vascular disease	HUVECs	H_2_O_2_	[98]
Heart	Myocardial infarction	H9c2 cardiomyocytes	Hypoxia/reoxygenation	[99]
Lung	Acute lung injury	Female C57BL/6 mice	LPS	[93]
Lung	Acute lung injury	Male BALB/c mice	LPS	[102]
Lung	Lung cell injury	A549 cells	*Staphylococcus aureus*	[103]
Lung	Inflammatory	NCI-H292 cells	PMA	[104]
Liver	Hepatotoxicity	Kunming mice	Acetaminophen	[105]
Liver	Oxidative damage	Male Wistar rats	AS_2_O_3_	[106]
Liver	Hepatic steatosis	C57BL/6N mice	High-fat diet	[107]
Liver	Acute liver injury	Male ICR mice	LPS/D-GALN	[108]
Liver	Non-alcoholic fatty liver	Male mice	High-fat diet	[109]
Kidney	Kidney injury	Male BALB/c mice	Cisplatin	[110]
Kidney	Nephropathy	Male Wistar-Kyoto rats	Doxorubicin	[111]

**Table 6:** Anti-cancer effects of eriodictyol.

Type	Experimental Subject	Theory or Pathway	References
Hepatocellular cancer	HepG2	Associated with cell cycle arrest and modulation of apoptosis-related proteins	[112]
Hepatocellular carcinoma	HepG2 and Huh-7 cells	NLRP3 inflammasome inactivation	[113]
Colorectal adenocarcinoma	-	It had a high degree of interaction with apoptotic proteins	[114]
Colorectal cancer	Male albino Wistar rats	Inhibition of carcinogens and the occurrence of aberrant crypt foci, regulation of lipid peroxidation markers and biological invertase, maintenance of antioxidant defense enzymes	[115]
Colorectal cancer	Human colon epithelial cell line and human CRC cell line	Inhibited fucosylation by down-regulating TSTA3 expression thus suppressed EMT process.	[116]
Lung cancer	A549 human lung cancer cell line	Induced mitochondrial apoptosis, G2/M cell cycle arrest and inhibited the mTOR/PI3K/AKT cascade	[117]
Nasopharyngeal cancer	CNE1 cancer cells	MEK/ERK signaling pathway	[118]
Gastric cancer	Gastric cancer cells	PI3K/AKT pathway	[119]
Ovarian cancer	A2780 and CaoV3	Nrf2/HO-1/NQO1 signaling pathway	[120]

## Abbreviation

4HPA3H	4-hydroxyphenylacetate 3-hydroxylase
AD	Alzheimer’s disease
AKT	Protein kinase B
APP	Amyloid precursor protein
ARE	Antioxidant response element
BAX	Bcl-2-related X protein
Bcl-2	B-cell lymphoma-2
cAMP	Cyclic adenosine monophosphate
COX-2	Cyclooxygenase-2
DSS	Dextran sodium sulphate
eNOS	Endothelial Nitric oxide synthase
ERK	Extracellular regulated protein kinases
D-GALN	D-galactoamine
GC-MS	Gas chromatography–mass spectrometry
GRAS	Generally regarded as safe
HO-1	Heme oxygenase-1
HPLC	High-Performance Liquid Chromatography
ICAM-1	Intercellular adhesion molecule-1
ICR	Institute of Cancer Research
IL	Interleukin
LC-DAD-MSD	Liquid chromatography serially coupled to diode array and mass selective detection
LC-MS/MS	Liquid chromatography tandem mass spectrometry
LPS	Lipopolysaccharide
MAPK	Mitogen-activated protein kinase
MEK	External signal-regulated kinase
MIP-1	Macrophage inflammatory protein-1
mTOR	Mammalian target of rapamycin
NFκB	Nuclear factor kappa-B
NLRP3	NOD-like receptor pyrin domain-containing 3
NMR	Nuclear magnetic resonance spectroscopy
NQO1	NADH dehydrogenase quinone 1
Nrf2	Nuclear factor erythroid 2
PI3K	Phosphoinositide 3-kinase
PKA	Protein kinase A
PS1	Presenilin 1
PSE	Ethanol extract of peanut shells
ROS	Reactive oxygen species
TGF-β	Transforming growth factor-β
TNBS	Trinitro-benzene-sulfonic acid
TNF-α	Tumor necrosis factor-α
TRP	Tyrosinase-related protein
UHPLC-ESI-MS	Liquid chromatography electrospray ionization tandem mass spectrometry
UPLC-Q-Exactive Orbitrap	Ultra-high-performance liquid chromatography coupled with quadrupole electrostatic field orbitrap high-resolution
UVA	Ultraviolet A
VEGR	Vascular endothelial growth factor
